# On the Origin of Superoxide Dismutase: An Evolutionary Perspective of Superoxide-Mediated Redox Signaling

**DOI:** 10.3390/antiox6040082

**Published:** 2017-10-30

**Authors:** Adam J. Case

**Affiliations:** Department of Cellular and Integrative Physiology, University of Nebraska Medical Center, 985850 Nebraska Medical Center, Omaha, NE 68198, USA; adam.case@unmc.edu; Tel.: +1-402-559-3078

**Keywords:** redox biology, redox signaling, oxidative stress, reactive oxygen species, hydrogen peroxide, oxygen, nitric oxide, evolution, great oxidation event, adaptation, metabolism, iron, copper, zinc, manganese, nickel

## Abstract

The field of free radical biology originated with the discovery of superoxide dismutase (SOD) in 1969. Over the last 5 decades, a plethora of research has been performed in species ranging from bacteria to mammals that has elucidated the molecular reaction, subcellular location, and specific isoforms of SOD. However, while humans have only begun to study this class of enzymes over the past 50 years, it has been estimated that these enzymes have existed for billions of years, and may be some of the original enzymes found in primitive life. As life evolved over this expanse of time, these enzymes have taken on new and different functional roles potentially in contrast to how they were originally derived. Herein, examination of the evolutionary history of these enzymes provides both an explanation and further inquiries into the modern-day role of SOD in physiology and disease.

## 1. Introduction

Life is derived by the movement of electrons [1]. Our existence revolves around the constant acquisition of electrons from the environment, which are then exploited to drive energy production by their passage down reduction potentials. Molecular oxygen (O_2_) has demonstrated itself to be an ideal electron sink, and thus, is represented across biology as a final electron acceptor in oxidative metabolism. However, the use of O_2_ in biological systems with high electron fluxes comes at the potential threat of generating reactive oxygen species (ROS), which may damage vital cellular components. Ground state O_2_ possesses two unpaired electrons with parallel spins in opposing orbitals, which under standard biological conditions energetically favors the reduction of O_2_ by one-electron transfers [2]. This one-electron reduction of O_2_ initially leads to the formation of superoxide (O_2_•^−^), which has been estimated to exist in biological systems at concentrations ranging from 10 to 200 pM [2,3]. Due to this, the development of ROS-detoxifying enzymes proved necessary for primitive life to exist in an oxidative world, and one of the first of these antioxidant enzymes was known as superoxide dismutase (SOD). In this review, I present a unique evolutionary perspective into this class of enzymes in homage to the late Charles Darwin, who so eloquently examined species in a similar light [4]. In doing so, I present a comprehensive look into the origins of SOD, and how billions of years of evolution has altered its role in cellular signaling and function.

## 2. The Major Oxidation Events

The conditions of primitive earth existed in stark contrast to the environment of today. Over the approximate 4.5 billion year (Gyr) history of the planet, the compositions of both the atmosphere and oceans have significantly evolved from reducing to oxidizing. Ancient atmospheric components (which are major contributors of oceanic constituents) were determined largely by volcanic gasses that produced an abundance of hydrogen (H_2_), carbon dioxide and monoxide (CO_2_ and CO), hydrogen sulfide (H_2_S), methane (CH_4_), and many other reducing, inorganic compounds [5]. The switch to an oxidizing environment was driven primarily by the appreciable accumulation of O_2_, however, the specific timeline of this event is still debated. 

A relative consensus exists among paleontology researchers that places the first major accumulation of O_2_ approximately 2.4–2.1 Gyr ago [6]. The primary piece of evidence supporting this timeline is derived from examination into different isotopes of sulfur [7]. In our current oxidative atmosphere, sulfur isotopes (i.e., 33S, 34S, and 36S) exist in tightly controlled ratios dependent upon their specific mass. However, Archean sediment deposits were shown to have a predominance of non-oxidized sulfur deposits (like pyrite) which contained differential ratios of sulfur that were independent of their mass [7]. These mass independent fractionations (MIF) of sulfur are not well understood, but thought only to occur through extrinsic forces such high energy ultraviolet and photochemical dissociation, which could occur in an atmosphere lacking significant quantities of oxygen and a protective ozone layer [8]. Sediments older than 2.4 Gyr possessed high MIF values while those younger than 2.1 Gyr were low [7]. Taken together with the increased abundance of oxidized species of sulfur after 2.1 Gyr, the data are highly suggestive of an increase in atmospheric O_2_ greater than 10–5 of present atmospheric levels (PAL; which has been estimated to be the minimum amount of O_2_ to significantly impact MIF values [9]) that occurred during this ancient time frame known as the Great Oxidation Event (GOE) [5].

While MIF sulfur isotope evidence strongly argues for a major oxidation event between 2.4 and 2.1 Gyr ago, other dating methodology suggests slightly variable timeframes for this first major accumulation of O_2_. For example, Anbar et al. identified the presence of highly redox sensitive transition metals in 2.5 Gyr ancient sediments [10]. The observation of these metals in ancient ocean sediment are highly suggestive of their transition from crustal rocks into the oceans, which would only occur during oxidative weathering in the presence of appreciable O_2_ levels. If this is indeed true, then oxidation of the atmosphere could have occurred earlier than sulfur MIF data would suggest. In another example, Frei et al., propose significant O_2_ accumulation even earlier than 2.5 Gyr by the use of chromium isotopes and oxidation states [11]. In current atmospheric conditions (i.e., elevated O_2_), chromium is rapidly oxidized from a +3 state to a +6 state by the presence of manganese oxides. Conversely, when O_2_ is low manganese oxides are scarce, and thus chromium predominantly resides in the +3 state. Moreover, in low O_2_ conditions reduced iron is readily available which is able to convert any +6 chromium back to the +3 state. When ocean sediments were examined for chromium, an abundance of +6 chromium was discovered dating between 2.8 and 2.6 Gyr ago, which suggests O_2_ accumulation even earlier than current theories [11]. Taken together, while the exact dates of O_2_ appearance are debated, the observation of O_2_ accretion ranging somewhere between 3.0 and 2.0 Gyr is not disputed.

The GOE was the first major event to increase atmospheric and oceanic O_2_ levels. However, the majority of researchers only believe this event to have increased O_2_ levels to 0.1–15% PAL [12]. A second oxygenation event known as the Neoproterozoic Oxidation Event (NOE), which occurred between 0.8 and 0.5 Gyr ago, is suggested to have been the second major oxidation incident that elevated O_2_ levels to PAL [13]. Comparable to the methods discussed in identification of the GOE, similar atomic findings are highly suggestive of even greater O_2_ levels during the NOE. These methods include decreasing cerium values [14], enriched molybdenum in black oceanic shales [15], changes in sulfur isotope and oxidation ratios [16], and differential chromium oxidation states [11]. Interestingly, many researchers have reported that the time frame between the GOE and NOE (approximately 1.9–0.9 Gyr ago) demonstrated a constant and unchanging pattern of atomic isotopes and redox states [12]. As such, this period has been referred to as the “boring billion” due to its apparent unremarkably stable atmospheric and oceanic O_2_ content [13], though this view has been contested [6,11]. Together, the GOE, boring billion, and NOE present a well-accepted two-step transition from a virtually anoxic environment to present day conditions. This view has been challenged recently by Lyons et al., who presents a more gradual increase in O_2_, which he terms the “Great Oxidation Transition (GOT)” [6]. This hypothesis of steady increases in O_2_ combined with chaotic fluctuations of O_2_ sources and sinks is gaining significant traction in the field as it accounts for data that currently does not fit within the standard two step model [6,17]. While models and specifics are still debated, one aspect of the atmosphere progression from reducing to oxidizing is consistently acknowledged: the primary source of molecular O_2_ that engulfed the earth derived from enzymatic processes involved with oxidative photosynthesis [18]. 

## 3. The First Enzymatic Processes

It is highly likely that simple proteins and enzymes evolved prior to nucleic acids, and as such were critical in establishing vital cellular processes like photosynthesis, metabolism, and replication. Hallmarks of most enzymatic systems are metals used for either protein stability or catalysis. The exact metal utilized by an enzyme system is constrained by two primary factors: the bioavailability of the metal and the utility of the metal for its designated purpose. In some instances, utility of metals may supersede limited bioavailability, however, readiness of specific metals appears to be the driving force behind most of enzyme evolution [19].

As previously discussed, primitive earth existed in a strong reductive environment with minimal molecular O_2_, which made the ancient oceans either anoxic or euxinic (anoxic and sulfidic) [20]. High sulfur (S) content significantly affects metal solubility with iron (Fe), nickel (Ni), and manganese (Mn) dissolving predominantly under these conditions, while metals such as zinc (Zn) and copper (Cu) precipitate and are biologically inert [19]. In contrast, under present day O_2_ conditions bioavailability of these metals is completely reversed, known as the Irving-Williams series of metal stability [21,22]. Thus, it is hypothesized that the environmental conditions of primitive earth provided the key elements to create the first enzymes, which by no coincidence contain a preponderance of the most abundant elements at that time: Fe and S. In the absence of O_2_, Fe and S have been shown to self-assemble into clusters that form the catalytic basis of the oldest known enzymes [23,24]. Fe in particular may be the most important element in the origin of life due to its ability to act as both an electron donor and acceptor as it cycles between its oxidized (+3) and reduced (+2) states. The utility of Fe is irrefutable, as even in present day O_2_ levels where Fe solubility and bioavailability are low, countless enzymatic processes rely on this heavy metal for critical life processes. 

One major physiological pathway that has relied on Fe since its origin is photosynthesis. Photosynthesis evolved as a method of long-term energy storage in the form of electron rich organic compounds, which would be essential for sustained life. This process is achieved by the systematic passage of electrons up their electrochemical gradients by the use of redox couples and energy from sunlight. Fe’s bioavailability on ancient earth combined with its versatility as a redox couple proved essential in the evolution of this process. While modern-day oxidative photosynthesis utilizes water (H_2_O) as an electron donor, it is believed primitive photosynthesis evolved from green/purple sulfur bacteria that could have utilized S, H_2_, and/or H_2_S as the original electron donors [25]. Interestingly, while variations in photosynthesis still occur today, the enzymes involved in all forms of this metabolic process are virtually identical and include Fe-containing proteins such ferredoxin, ribulose-1,5-bisphosphate carboxylase/oxygenase (RuBisCO), and Fe-S containing photosystems [26]. This suggests these Fe-containing enzymes evolved long before the transition to oxygenic photosynthesis, and their widespread existence in the oxidative and limited free-Fe world of today implies the importance of this metal in their functionality. 

The timeline for the appearance of oxidative photosynthesis is highly debated in similar fashion to the GOE. Estimates range from 3.7 to 2.3 Gyr ago depending upon the methodology utilized and inferences made from oxidative and isotopic evidence [27,28]. The most widely accepted evidence of oxidative photosynthesis appears around 2.7 Gyr ago in the form of fossilized sterane in ancient sediments [29]. Sterane is a form of sterol, an organic molecule that is derived from cholesterol and found in cell membranes, and requires molecular O_2_ for its production. The presence of sterols in ancient fossilized cyanobacteria, which are modern-day oxidative photosynthesizers, suggests the presence of appreciable O_2_ inside these cells at least 300 million years prior to the accepted start of the GOE. These observations have led to the conventional hypothesis that oxidative photosynthesis predated the GOE, and is the single greatest contributor to the development of an O_2_-rich atmosphere and oceans. However, this premise is highly counterintuitive when taking into account that O_2_ is a highly reactive and oxidizing molecule that would have been toxic to primitive life evolving in a reducing environment. It would be supposed that an over-production of O_2_ waste would have eventually caused widespread extinction of ancient life, but this is clearly not the case. Through what could be considered one of the most extreme evolutionary pressures ever to exist on earth, life evolved ways of counteracting O_2_ toxicity, which have proven quintessential for existence in an oxidative world.

## 4. The First Antioxidants

Molecular O_2_ possesses two p-orbital unpaired electrons that exist in the same spin state, which makes ground-state O_2_ both paramagnetic and highly susceptible to univalent reduction [30]. Due to this oxidizing capacity (the term “oxidizing” originated from reactions with O_2_), O_2_ serves as an excellent terminal electron acceptor for many biological processes such as oxidative phosphorylation in the mitochondria to generate water. While beneficial for many molecular pathways, the oxidizing nature of O_2_ may also contribute to the uncontrolled pathological removal of electrons. This not only damages vital cellular components (i.e., nucleic acids, proteins, lipids, and sugars), but the addition of these electrons to O_2_ creates reactive species far more damaging than ground-state O_2_ (i.e., ROS). These ROS exist in many varieties, however, the primary species derived from univalent reduction of O_2_ is superoxide (O_2_•^−^). O_2_•^−^ is a reactive free radical capable of directly or indirectly deriving most major ROS and free radical species that exist in biological systems. Thus, early life existing in the presence of appreciable O_2_ would have a strong evolutionary advantage if they were able to eliminate this linchpin ROS.

While the number and diversity of species exponentially expanded around the major oxidation events (i.e., GOE and NOE), fossil records suggest that the earliest forms of life arose over 4.0 Gyr ago, which is greater than 1.5 billion years before the accepted beginning of the GOE [31]. Modern day sequencing and phylogenetic analyses have allowed for a deeper understanding into the evolution and diversification of species and enzymes from these primitive life forms. Although it may be intuitive to assume that antioxidants (i.e., ROS detoxifying molecules and enzymes) evolved during the GOE to counteract the oxidative pressure of accumulating O_2_, sequencing analyses of ancient species suggest antioxidants originated long before O_2_ was abundant in the atmospheres and oceans. This is most likely due to the localized formation of O_2_ due to either abiotic sources (e.g., photolysis of H_2_O by ultraviolet light) or cohabitation in close proximity to an oxidative photosynthesizing organism. 

Exhaustive sequencing, alignment, and phylogenetic analyses have been performed examining primitive antioxidant systems [32,33,34,35,36,37,38,39,40,41,42]. It is clear from these studies that three primary ROS removal enzymes existed prior to the GOE: SOD, catalase (CAT), and peroxiredoxins (PRDX; previously known as thioredoxin peroxidases). SOD is aptly named as its only known function to date is the dismutation of O_2_•^−^ to hydrogen peroxide (H_2_O_2_). Similarly, CAT’s and PRDX’s singular roles are the decomposition of H_2_O_2_, though different mechanisms are used to achieve the same endpoint. Together, these enzymes act to remove O_2_•^−^ derived ROS from the biological system, which in primitive, simple lifeforms was essential to counteract the toxicity of O_2_ derived reactive species. As previously discussed, at the time life arose the oceans were highly euxinic, which made Fe and S highly bioavailable. Due to this, the most primitive form of SOD used Fe at its catalytic center, CAT also utilized Fe for its activity, and PRDX exploited the use of S-rich thiols to detoxify H_2_O_2_. Interestingly, while CAT and PRDX have not changed significantly since ancient times and still rely on Fe and S for their activities, SOD has evolved various heavy metal isoforms and subcellular compartments dependent upon the complexity of the organism.

## 5. Superoxide Dismutase Evolution

Currently, there are three discrete families of SOD that all perform the same reaction: 2O_2_•^−^ + 2H^+^ → H_2_O_2_ + O_2_. The SOD families are defined by the metals utilized for stability and catalysis as well as the overall structure of the enzymes. Each of the families contains various isoforms of the enzyme, and possesses the following heavy metals: Fe or Mn, Cu and Zn, or Ni. The most primitive of the families is the Fe- or Mn-containing SODs. Fe-containing SOD (FeSOD) is believed to be the most ancient form of SOD, and is still one of the most common forms of SOD in various modern-day strains of both aerobic and anaerobic bacteria to date [43,44]. Interestingly, FeSOD has also been identified in Archaea [45], Protists [46], and even eukaryotic plants [47,48] providing evidence not only to the prolonged existence of this SOD isoform, but also the versatility. The other isoform of SOD in this family contains Mn (MnSOD), and is also found in all species ranging from Archaea to Eukarya. FeSOD and MnSOD possess close to 50% sequence homology with virtually identical active sites [49,50], suggesting the two enzymes are closely related and evolved from a close common ancestor. This relation is further supported by the identification of specific forms of SOD termed cambialistic (meaning “exchange” in Latin) that are able to utilize either Fe or Mn in their active site dependent upon metal availability [51,52,53]. These cambialistic SODs may either be the “missing link” between the evolution of FeSOD and MnSOD, or may simply serve as a new evolutionary branch generated due to the need for versatility of metal availability. In either event, FeSOD and MnSOD appear to have originated in very early in primitive lifeforms, and the metals utilized were both highly bioavailable (due to low atmospheric and oceanic O_2_ levels) and efficient catalytic centers for the dismutation of O_2_•^−^. The two enzymes, however, do possess significant differences in their oxidation and reduction potentials [54]. Over time, this could have provided significant advantages to organisms in variable O_2_ and heavy metal niches, and may explain the persistence of both isoforms in various species today.

The second family of SODs possesses both Cu and Zn (CuZnSOD), and is believed to be the most modern family of the SOD lineages. The CuZnSOD’s are ubiquitous among plant and animal species, and are localized to the nucleus, cytoplasm, mitochondrial intermembrane space, chloroplast, and even the extracellular matrix [37,55,56,57]. This class of SOD is also found in bacteria, however, it is expressed in much lower levels and confined to the periplasmic space in only select gram-negative bacteria [58]. CuZnSOD is not found in archaeal or protist genomes [59,60], suggesting this isoform developed at a much later time point in evolution than FeSOD or MnSOD. An additional clue as to its late evolutionary origins is that CuZnSOD likely evolved during or after the GOE as both Cu and Zn bioavailability increases significantly with enhanced atmospheric and oceanic O_2_ levels [19]. Interestingly, the CuZnSOD family shares minimal structural homology with the Fe/MnSOD family signifying these enzymes were derived independent of one another and underwent convergent evolution to perform identical reactions in removing O_2_•^−^ from a biological system [37]. Even more striking is that even within the CuZnSOD family, the extracellular form found in higher-level eukaryotes more closely resembles that of fungi than the intracellular eukaryotic form [37]. This suggests either the extracellular form of CuZnSOD may represent a more primitive version from which the intracellular version divergently evolved, or possibly both enzymes converged on enzymatic reactions as well as metals utilized for stabilization and catalysis. 

The last family is the Ni-containing SODs (NiSOD). These isoforms are primarily contained to marine bacteria and algae [42,61]. Compared to the exhaustive studies performed on the other families of SOD, much less is known about NiSOD due to its comparatively recent discovery in 1996 [62]. Unlike the dimeric and tetrameric forms of the other SODs, NiSOD functions as a homohexamer that is structure is not dependent upon Ni coordination [63]. From an evolutionary standpoint, NiSOD does not appear structurally related to the other families of SOD, and may again represent another example of convergent evolution for O_2_•^−^ removal. Additionally, due to its predominant presence in marine species, it is theorized that NiSOD evolved around the time of GOE in response to the decreasing bioavailability of Fe in the oceans due to increased free O_2_ produced from oxidative photosynthesis [42]. 

To date, the SODs are the only known class of enzyme able to autonomously eliminate O_2_•^−^ in a biological system. Another class of O_2_•^−^ removal enzymes, known as the O_2_•^−^ reductases (SOR), has been identified in Archaea, Bacteria, and unicellular eukaryotes [64]. Similar to NiSOD, far less is known about these enzymes due to their relatively recent discovery [65]. The mechanism, however, of SORs is well established: O_2_•^−^ + 2H^+^ + e^−^ → H_2_O_2_. In contrast to the SODs, SORs are only able to reduce 1 molecule of O_2_•^−^ per reaction, do not produce molecular O_2_, and require the addition of an electron provided from a reducing equivalent (i.e., reduced forms of nicotinamide adenine dinucleotide or nicotinamide adenine dinucleotide phosphate). All SORs contain Fe at their active site, are present in Archaea species, and possess minimal homology to modern-day SODs, which may suggest SORs are primitive forms of O_2_•^−^ removal in ancient species. Conversely, the presence of SORs in modern-day species (alone or in combination with SOD) suggests these enzymes may provide an evolutionary advantage. While requiring reducing equivalents to eliminate O_2_•^−^ would limit the availability of these electrons to be used for energy production, the fact that SORs do not produce molecular O_2_ during the removal of O_2_•^−^ may be vitally important to certain anaerobic organisms. Speculations such as this would infer that SORs may have evolved independently of SODs as an alternative O_2_-deplete mechanism of O_2_•^−^ disposal. The history of the SORs is still very unclear, and warrants further investigations into the origins of these alternative O_2_•^−^ elimination enzymes.

While the evolutionary histories of all of these enzymes both diverge and converge, all organisms to date have been shown to possess some form of O_2_•^−^ removal system. This observation was at one time not fully accepted, as it was believed that certain species such as Neisseria gonorrhea, Mycoplasma, *Lactobacillus plantarum*, and even mammalian adipocytes did not possess any form of SOD or SOR [66]. All of these, with the exception of *Lactobacillus plantarum*, have now been shown to possess some form of SOD [67,68,69]. The absence of SOD from *Lactobacillus plantarum* does not invalidate the O_2_•^−^ theory of O_2_ toxicity, as these organisms (as well as Neisseria gonorrhea) have evolved the ability to concentrate high levels of free Mn in their cytoplasm, which acts as a O_2_•^−^ scavenging system independent of enzymatic activity [67,70]. Intriguingly, certain viruses have also demonstrated genetic encoding of various SOD isoforms [71,72,73]. It is unclear if the expression of SOD in a host organism provides the virus with an evolutionary advantage, or if the presence of SOD genes in the viral genome is simply an example of lateral gene transfer from a previous host. This concept of lateral gene transfer extends beyond viruses into all known SOD-possessing organisms when examining the evolution of these O_2_•^−^ removal enzymes. While comprehensive sequencing of modern-day organisms has greatly expanded our understanding of how genes have evolved from ancient species, it is virtually impossible to know by sequencing alone if genes were simply transferred from one organism to another as opposed to the true evolution of a new protein derived from a previously existing one.

Regardless of the exact evolutionary lineages of SOD, the necessity of O_2_•^−^ scavenging in an O_2_-rich environment to prevent oxidative damage is undeniable. However, when examining the function of SOD during evolution one question remains: Why is one of the major products of SOD a potentially harmful ROS (i.e., H_2_O_2_)? While multiple H_2_O_2_ detoxifying systems exist in most organisms (i.e., glutathione peroxidases, thioredoxins, catalase, etc.), it remains unclear why after billions of years of evolution that no single enzyme exists that is able to convert O_2_•^−^ directly to less reactive molecules like water and/or O_2_. Logic would dictate that this hypothetical enzyme would negate the need for multiple redundant H_2_O_2_ removal systems, which would relinquish a significant amount of biological resources and energy that could be utilized elsewhere in the organism. While a single enzyme ROS removal system could potentially be a weakness to an organism by “putting all the eggs in one basket,” this vulnerability could easily be counteracted by the implementation of various redundant isoforms, splice variants, and subcellular locations. Understanding that evolution is purely driven by selectively advantageous variations, it puts forth the argument that generating H_2_O_2_ from O_2_•^−^ may be beneficial from an evolutionary standpoint, but the question still remains: Why?

## 6. Early Utilization of ROS

The passage of electrons for the purpose of energy generation lies at the epicenter of life. Organisms have evolved various methods of extracting and utilizing electrons from their surroundings to fulfil this energy demand. In a perfectly efficient system, electrons are eventually deposited on a terminal acceptor that generates an inert chemical species to be utilized elsewhere in the organism. However, biology is far from perfectly efficient, and a large flux of electrons creates the potential for non-directed escape of these charged particles onto undesired target molecules. Interestingly, O_2_ may act in both capacities as a 4 electron terminal acceptor to form inert water, or the unsought 1 electron target to form O_2_•^−^. Early in evolution, O_2_ was most likely not the preferred terminal acceptor for general metabolism as it was only created from localized abiotic sources in very low quantities. As oxidative photosynthesis became the preferred method of long term electron storage for energy production, the amount O_2_ gradually increased. O_2_, being a di-radical species, makes it the perfect electron acceptor to form inert water, and as such it is no surprise that O_2_ was eventually assimilated into metabolism as a critical component to electron movement. However, O_2_’s potential for reactivity and ROS generation created a Janus-faced situation in which cells had to rapidly adapt. 

Early ROS scavenging was essential to life. As such, organisms that possessed mechanisms to eliminate radical and reactive species proved evolutionarily advantageous, and thus ROS elimination methods were propagated in future generations. With ROS being an assured outcome in an O_2_-rich environment, it is also logical that organisms that adapted to harness these reactive molecules may be at an even further advantage on the evolutionary scale. Mittler and colleagues have put forth a hypothetical model describing the utilization of ROS for signaling and protection as it pertains to the advancement of a species [74]. First, ROS scavenging is serendipitously developed as a means of survival in primitive species. This is soon followed by adaptive processes in the cell allowing response and fine tuning of ROS scavenging. As an organism evolves, ROS produced from other organisms begins to allow cell-to-cell communication and sensing of the environment. Last, ROS is purposefully generated within the organism by dedicated machinery for the purposes of protection, communication, and signal transduction. Each one of these steps would have provided primitive organisms’ advantages over their competition by exploiting the oxidative environment for their added survival. With O_2_•^−^ being the foundation of all O_2_-based ROS, it follows logically that cells adapted to the utilization of this specific reactive species to coordinate cellular activities. 

## 7. O_2_•^−^-Mediated Redox Signaling

A grey area exists between the terms “oxidative stress” and “redox signaling”. The former is often used to describe any process involving ROS, however, it is now well accepted that ROS regulate essential physiological processes that would not be considered stressful to the cell or organism. More refined definitions of these terms suggest that redox signaling encompasses the cellular use of ROS in a reversible manner to purposefully direct normal biological processes, while oxidative stress is the irreversible damage of cellular components due to an uncontrolled production of ROS. For example, an example of prototypical redox signaling would be H_2_O_2_-mediated oxidation of cysteine residues. Under certain concentrations of H_2_O_2_ (often sub-micromolar range, but this is dependent upon many factors such as cell type, age, species, etc. [75]) is able to oxidize the free thiol group of cysteine to various reversible oxidation states (similar to that of phosphorylation), which may then go on to form other modifications such as disulfide bridges, cross-links with glutathione, and other variations of covalent sulfur bonds that can affect protein function [76]. In the event H_2_O_2_ becomes too high in concentration (often high micromolar or millimolar range, but again is dependent on many variables [77]), the cysteine residues may become oxidized beyond a reversible state, and thus the protein becomes permanently damaged and possibly non-functional (i.e., oxidative stress). While these definitions appear clear from the outset, biology is highly dynamic and constantly adapting to all environmental changes, big or small. Thus, both low levels of ROS leading to “redox signaling” events as well as high levels of ROS causing “oxidative stress” will both trigger signaling cascades and adaptive processes within a cell. This begs the question, does oxidative stress even exist or is redox signaling simply the response to the entire spectrum of high and low levels of ROS?

An illustrative example would be O_2_•^−^-mediated oxidation of Fe-S cluster enzymes. O_2_•^−^ has been long established to readily oxidize Fe-S cluster enzymes at an incredibly fast rate [78]. Classic, 4Fe-4S clusters are the most sensitive to O_2_•^−^, and oxidation by O_2_•^−^ leads to an unstable 3Fe-4S form that is catalytically inactive [79]. Persistent levels of elevated O_2_•^−^ can further oxidize the cluster causing additional removal of Fe molecules leading to added destabilization of the complex. By many definitions, this would be considered oxidative stress due to the damaging inactivation of an enzyme. However, inactivation of these enzymes by O_2_•^−^ often triggers major cellular changes in response to their dysfunction caused by ROS. For example, mitochondrial aconitase and succinate dehydrogenase are two Fe-S cluster enzymes that participate in the citric acid cycle in the matrix of the mitochondria, and both have been shown to be highly susceptible to inactivation by O_2_•^−^ [80,81]. Aconitase inactivation by O_2_•^−^ leads to a build-up of citrate, which is then often diverted into fats for long-term energy storage [82]. Succinate dehydrogenase inactivation by O_2_•^−^ leads to the build-up of succinate, which is a product inhibitor of the prolyl-hydroxylase family of enzymes that negatively regulate the hypoxia inducible factor 1 alpha (HIF1α) [83]. Thus, inactivation of succinate dehydrogenase stabilizes HIF1α, which promotes more glycolytic activity. In both of these situations (i.e., aconitase and succinate dehydrogenase inactivation), the cell has diverted its activities away from the mitochondrial oxidative metabolism in response to elevated levels of mitochondrial O_2_•^−^, which often increases during times of mitochondrial dysfunction or excess electron buildup. This example depicts the cell’s ability to exploit specific subcellular levels of ROS as a readout of cellular function and adapt accordingly to benefit the cell. This advantageous usage of ROS could be considered a form of oxidative hormesis [84], and in fact has recently been described more accurately as oxidative eustress [85]. This furthers the concept that oxidative stress (or oxidative distress) is a term that should be reserved only to the rare situation in which ROS exceed the physiological capacity of a cell to respond leading to pathophysiological effects. Regardless of semantics, the ability of O_2_•^−^ to affect cellular processes in irrefutable. However, O_2_•^−^ is a highly reactive molecule with a short half-life, which makes its versatility as a signaling molecule limited. The conversion of O_2_•^−^ to H_2_O_2_ creates a non-radical and stable ROS capable of delivering the oxidative message over a longer cellular distance, and as such the rapid conversion of O_2_•^−^ to H_2_O_2_ most likely proved to be a selective benefit during evolution as species evolved to utilize ROS to their advantage.

## 8. SOD-Mediated Redox Signaling

After billions of years of evolution, modern-day organisms have developed machinery capable of utilizing O_2_•^−^ as a signal transducer. However, to primitive life O_2_•^−^ was likely nothing more than an O_2_-derived toxin capable of causing extensive redox damage to vital cellular components. The ability to detoxify O_2_•^−^ rapidly would serve as a robust selective advantage during the course of evolution, and the occurrence of SOD early in the history of life strongly supports this sentiment.

Aforementioned, both ancient and modern forms of SOD all possess the same mechanism of converting 2O_2_•^−^ and 2H^+^ into O_2_ and H_2_O_2_. The perplexing generation of two oxidizing species from O_2_•^−^ suggests one (or a combination) of three possibilities: (1) these oxidants were somehow beneficial to primitive life in a highly reductive environment; (2) primitive life already possessed additional detoxification methods for these oxidants; or (3) O_2_ and H_2_O_2_ still were highly toxic to primitive life, but less reactive than O_2_•^−^ (i.e., lesser of two evils). The latter is the most likely possibility, but the H_2_O_2_-removal enzymes catalase and PRDX have been dated to exist in primitive life inferring the possibility of additional antioxidant systems co-evolving in a similar timeframe [41]. Moreover, the utilization of H_2_O_2_ by primitive peroxidase systems to generate reactive intermediates (i.e., hypochlorous acid, hypothiocyanous acid, etc.) may have served as a primitive defense system, however, this is purely speculative. In any event, the production of H_2_O_2_ is a hallmark of all O_2_•^−^ removal systems throughout evolutionary history, which suggests enzymes like SOD play a key regulatory role in the production of this non-radical ROS.

A longstanding theory states that the amount of SOD within a system is directly correlated to the quantity of H_2_O_2_ produced [86], but this paradigm significantly minimizes the complexity of O_2_•^−^ derived cellular reactions. Liochev and Fridovich propose a more dynamic theory of H_2_O_2_ production from SOD that is situation-dependent [87]. The authors describe three situations in which the addition of SOD could affect steady-state cellular H_2_O_2_ levels. The first is the simplest situation where 100% of O_2_•^−^ spontaneously dismutates to H_2_O_2_ at the estimated rate of 10^5^ M^−1^ s^−1^. In this case, the addition of SOD would only expedite the conversion of O_2_•^−^ to H_2_O_2_, which would only appreciably decrease the steady state of O_2_•^−^ in the process. If H_2_O_2_ could not be effectively removed and O_2_•^−^ continually added to the system then over time H_2_O_2_ would appreciably accumulate faster in the presence of SOD. However, in a biological system H_2_O_2_ is constantly being removed by various H_2_O_2_ detoxifying enzymes systems, and as such no appreciable increase in H_2_O_2_ would be noted under these conditions. The second situation to consider would be if O_2_•^−^ was readily reacting with other cellular components to generate other non-H_2_O_2_ oxidation products prior to spontaneously dismutating to H_2_O_2_. If SOD was added to this system, the SOD would outcompete the other cellular components for O_2_•^−^ thus leading to an increased flux of H_2_O_2_ into the system. This is the only situation in which an increase in SOD could increase steady-state H_2_O_2_, but again, it would be staunchly dictated by the presence and kinetics of H_2_O_2_ removal enzymes as well. The last situation would be if O_2_•^−^ was readily being reduced to H_2_O_2_ by an external reductant (i.e., SOR). If SOD was added to this system, the production of H_2_O_2_ would be reduced by half since SOR generates one H_2_O_2_ for every O_2_•^−^, while SOD requires two O_2_•^−^ for each H_2_O_2_ produced. Taken together, SOD does not appear to have evolved to affect the quantity of H_2_O_2_ produced from O_2_•^−^, but moreover, the removal of O_2_•^−^ and/or the fine tuning of other oxidants.

Under the vast majority of circumstances, SOD effectively lowers the steady-state levels of O_2_•^−^ at an incredibly fast rate (10^9^ M^−1^ s^−1^) [87]. This diffusion-limited rate posits the possibility that removal of O_2_•^−^ is more important to vital cellular processes than is the production of H_2_O_2_. A classic theoretical example of this phenomenon in the preservation of nitric oxide (•NO) by SOD [88]. O_2_•^−^ and •NO non-catalytically combine to form peroxynitrite (ONOO^−^). Unlike •NO, ONOO^−^ is highly reactive and damaging, and is not believed to act in a coordinated manner as a signaling molecule. In theory, the addition of SOD would outcompete •NO for O_2_•^−^, thus preserving the bioavailability of •NO as a signaling molecule. While this theory makes intuitive sense, the situation is more complex than this (similar to that of the production of H_2_O_2_ by SOD) [89] and may only occur under certain biological conditions. Another example of SOD protecting another biological process from the effects of O_2_•^−^ would be in maintaining the reduced state of Fe. Heme formation occurs in the mitochondria, and the last step in this process requires reduced Fe to be inserted into the porphyrin ring. Our group has shown that the loss of MnSOD leads to an increased pool of oxidized iron, which ultimately causes dysfunctional heme synthesis and a porphyria-like phenotype [81]. While SOD may have eventually evolved to affect numerous signaling pathways, the preservation of reduced Fe in primitive life may have been one of its most important evolutionary functions that has persisted even in modern species today. 

The question still remains that if SOD’s primary function is to reduce steady-state O_2_•^−^ levels, then why is an oxidizing and reactive molecule (i.e., H_2_O_2_) formed in the process? If this damaging molecule was truly detrimental to the cell, it would be speculated that the mechanism of SOD would have rapidly evolved away from the formation of H_2_O_2_ into something more inert. The persistence of the H_2_O_2_-forming mechanism among all convergent forms of SOD suggests that both the lessening of O_2_•^−^ as well as production of H_2_O_2_ are beneficial for normal physiological processes. Aforementioned, under most biological situations SOD does not appear to affect steady-state levels of H_2_O_2_, so it could be postulated that SOD may affect the spatial orientation of this ROS. For example, CuZnSOD has been shown to be able to physically bind to Rac1, which is a subunit regulating specific isoforms of NADPH oxidases (NOX) [90]. NOX enzymes are professional O_2_•^−^ generating enzymes, suggesting the localization of CuZnSOD in close proximity to these proteins creates the opportunity to limit the diffusion distance of O_2_•^−^ to be converted to H_2_O_2_ in a tightly regulated and spatially oriented manner. Moreover, H_2_O_2_ derived from this NOX-CuZnSOD interaction has been directly implicated in the physiological activation of redox-sensitive transcription factors such as the nuclear factor kappa light chain enhancer of activated B-cells (NFκB) [91]. CuZnSOD has also been shown to be recruited to T-lymphocyte receptors during times of activation [92]. Understanding that NOX-derived O_2_•^−^ is critical in the activation of these immune cells [93], it once again infers that rapid and localized conversion to H_2_O_2_ is critical in propagating this ROS signal for normal physiological T-lymphocyte activation. Other forms of SOD have also been demonstrated to play a role in localizing H_2_O_2_-mediated signaling cascades in other physiological processes [94,95,96,97,98,99,100]. In summary, it appears that the ability of lowering O_2_•^−^ while concurrently producing H_2_O_2_ has proven to be advantageous in regards to both cellular viability and signaling in an oxidative environment. While in primitive lifeforms the role of SOD may have been relatively straight-forward, the complexity derived from billions of years of evolution has given SOD multifaceted roles in normal mammalian physiology as well as disease that are still be elucidated today.

## 9. SOD and Disease

Within mammals, SOD has been exhaustively studied for its potential role in disease. Interestingly, the majority of mutations in SOD that are associated with disease retain catalytic activity. Due to the essential protective role SOD plays in an oxidative atmosphere, this suggests complete catalytic loss of SOD may be incompatible with life in an O_2_-rich environment, thus, inactive forms of SOD are very rarely represented in any known disease state. However, active forms of the three SOD isoforms have been associated with mammalian diseases of varying cellular diversity, which further adds to the complexity of this ancient class of antioxidant enzymes.

The most studied disease possessing a SOD mutation is amyotrophic lateral sclerosis (ALS), where CuZnSOD is the specific isoform associated with this malady. CuZnSOD mutations account for approximately 20% of familial forms of ALS (most forms of ALS are sporadic, with approximately 10% being familial in nature), and over 150 mutations of CuZnSOD have been identified in this disease [101]. Intriguingly, with the exception of rare mutant forms of CuZnSOD that render the enzyme inactive [102], the majority of CuZnSOD mutants in ALS possess equal or higher amounts of intrinsic activity [101]. This argues one of two things: (1) that CuZnSOD mutations leading to ALS are not dependent upon SOD activity and based more on specific properties of the protein (i.e., ability to for inclusion bodies or protein aggregates [103,104,105]) or (2) the mutations cause differential localization of CuZnSOD leading to aberrant or deficient ROS signaling cascades. Aforementioned, the addition of SOD to a system only increases H_2_O_2_ under specific conditions, which suggests ALS caused by CuZnSOD mutants possessing more catalytic activity are likely due to one of the previous hypotheses as opposed to toxicity due to an over-production of H_2_O_2_.

Another disease often associated with an imbalance in CuZnSOD is trisomy 21, or Down syndrome. As the name implies, individuals with trisomy 21 possess an extra copy of chromosome 21 in which CuZnSOD is encoded in humans. Paradoxically, patients with trisomy 21 demonstrate significant oxidative imbalances in their redox state [106], which would not be predicted with elevated levels of CuZnSOD. It is believed that the overt oxidative environment observed in trisomy 21 comes from a myriad of players such as over-expression of pro-oxidant genes, down-regulation of anti-oxidant genes, and mitochondrial dysfunction [107,108,109]. Intriguingly, the corneal disease keratoconus has also been linked to mutations in CuZnSOD and is highly prevalent in patients with trisomy 21 suggesting another possible link to dysregulated CuZnSOD activity and disease [110,111]. Last, several diseases have been linked to aberrant CuZnSOD expression levels as opposed to mutation. These include various forms of malignancies and cardiovascular diseases [88,112].

Mouse models of CuZnSOD loss have provided unexpected results in regards to the function of this antioxidant enzyme. Five variants of CuZnSOD knockout mice have been generated on various backgrounds to date, and all appear to have very similar phenotypes [113,114,115,116,117]. Surprisingly, CuZnSOD deficient mice are viable and indistinguishable from wild-type mice at young ages [113]. However, as CuZnSOD knock-out mice age they begin to succumb prematurely to various pathologies such as behavioral changes, progressive denervation of motor neurons, muscle wasting, decreased fertility, macular degeneration, and an increased incidence of various malignancies [113,114,115,116,117,118,119]. The protection of young animals to the loss of CuZnSOD is suggestive of compensatory mechanisms of O_2_•^−^ removal, however, these have not been reported [113]. An alternative explanation is that CuZnSOD is simply dispensable for normal development. Given that CuZnSOD evolved much later than other SOD isoforms (possibly after the GOE when Cu and Zn became bioavailable with elevated O_2_), it is possible that this isoform of the enzyme was assimilated less as a protective enzyme and more to fine tune intracellular ROS levels for signaling purposes. Thus, its loss in modern-day species does not insinuate imminent death, but instead, dysfunction later in life. 

EcSOD loss is also not embryonically lethal and does not shorten the lifespan of mice, but loss of this isoform does increase the sensitivity to other pathologies later in life similar to loss of CuZnSOD [120]. EcSOD knockout mice are more sensitive to hypoxia and hyperoxia, which under these conditions manifests as ventricular hypertrophy, hypertension, renal injury, emphysema, as well as corneal dysfunction [121,122,123,124,125,126]. EcSOD null mice have also been shown to have increased sensitization to adriamycin-induced nephropathy, bleomycin-induced bronchopulmonary dysplasia and pulmonary hypertension, as well as neurocognitive deficits after radiation [127,128,129]. Interestingly, conditional knockout of EcSOD in the lung of adult animals leads to imminent death due to severe pulmonary fibrosis and dysfunction without any additional challenges [130]. This suggests the possibility of compensatory mechanisms during embryonic loss of EcSOD that are not present in a fully developed adult. However, expression and catalytic levels of the other isoforms of SOD are not altered in any tissue with loss of EcSOD, which further convolutes these data and warrants further investigation into this phenomenon [120]. 

In humans, both mutation and expression levels of EcSOD are associated with disease, however, no complete loss of function mutants have been reported to date. One of the most prevalent mutations of EcSOD is known as R213G. This alteration causes a positively charged arginine located in the heparin-binding domain of the enzyme to be converted to a glycine, which leads to increased circulating levels and redistribution of EcSOD. The R213G mutation has differential effects as it has been demonstrated to exacerbate cardiovascular disease in humans and mice exposed to hypoxia, but may also protect humans from chronic obstructive pulmonary disease and mice from lipopolysaccharide and bleomycin-induced injury as well as asthma [131,132,133,134,135,136]. This observation suggests that the selection pressure to preserve this mutation may have an advantage in certain settings but may increase disease risk in others. Other polymorphisms in EcSOD have been shown to decrease lung function and increasing risk for emphysema, but it remains unknown how these non-coding region mutations affect EcSOD expression [137,138]. Our group has shown in both mice and humans that epigenetic repression and downregulation of EcSOD significantly affects various forms of cancer growth and metastasis [139,140,141,142]. EcSOD has been shown to play a role cardiovascular diseases as well [88]. Because EcSOD is also a Cu and Zn containing enzyme, is located extracellularly, is the youngest evolutionary form of SOD in mammals, and mice devoid of EcSOD are viable and healthy under normal conditions, it could be speculated that EcSOD evolved to regulate extracellular ROS signaling pathways as opposed to protect vital cellular functions.

The final and most ancient form of mammalian SOD, MnSOD, has proven to be the most critical to survival in an oxidative environment. Mice lacking MnSOD die shortly after birth (once exposed to atmospheric levels of O_2_) from overt O_2_ toxicity that presents as dilated cardiomyopathy and neurodegeneration [143,144]. Additionally, we and others have shown that conditional loss of MnSOD demonstrates inappropriate organ system development (albeit certain cell types are affected more than others) [80,81,145,146,147,148,149,150,151,152]. It has been proposed that MnSOD may act as a tumor suppressor gene [153], and mice heterozygous for MnSOD indeed demonstrate an increased incidence of certain types of cancers [154]. Conversely, we have demonstrated that complete loss of MnSOD may be protective against the development of certain cancers [155]. These conflicting results suggest that the tumor suppressing effect of MnSOD may be dosage dependent, and the complete loss of MnSOD leads to cell death opposed to cancer formation. Additionally, these findings may also infer that the loss of MnSOD does not play a role in the initiation of cancer, but may play a stronger role in the promotion or progression of an already formed malignancy.

Similar to the other isoforms of SOD, no mutation leading to the complete catalytic inactivation of MnSOD has been observed in any human disease to date. Active mutant forms of MnSOD have been associated with increased risk for certain chronic conditions such as diabetes, breast cancer, and prostate cancer [156,157,158,159]. Interestingly, reduced expression of MnSOD by altering transcription, abnormal epigenetic regulation, or loss of heterozygosity has also been shown to be associated to human cancers [160,161,162,163,164], which again supports the concept that MnSOD acts as a tumor suppressor gene. In contrast, certain types of cancers as well as advanced metastatic tumors display over-expressed levels of MnSOD [165,166,167], which supports the hypothesis that the expression level of MnSOD differentially regulates various stages of cancer progression [168].

Together, the SODs regulate various aspects of normal physiology (see SOD-Mediated Redox Signaling section), and altering their functional capacity may lead to significant pathology dependent upon the specific temporal and spatial factors involved. As discussed, a strong body of literature exists suggesting the SODs play a distinct role in cancer development and progression. This is highly counterintuitive when evaluating the evolutionary role for SOD in cellular survival. We and others have hypothesized that SOD evolved essentially to protect primitive life from the damaging effects of O_2_•^−^, which would in turn allow these original cells the ability to grow and divide. However, cancer appears to develop or progress (in part) due to the functional loss of SOD activity, and these malignant cells appear to exist in a perpetual state of increased levels of ROS. These two observations further the model by Mittler that over billions of years of evolution life has changed from simply eliminating ROS to utilizing it as a selective advantage [74]. By this logic, cancer may be thought of as simply an over-adaptation to ROS by causing cellular proliferation to remain dysregulated. Work by Goswami and colleagues supports this theory as they have shown that normal cellular proliferation is tightly regulated by a redox cycle that occurs within the cell cycle [169]. Moreover, they demonstrate that SODs are directly involved in the control of cellular proliferation, and that dysregulation of these enzymes leads to disruption of normal cell cycle progression potentially leading to cancer [170]. Overall, the study of how SODs regulate the redox environment and cellular signaling is still in its infancy, and future research is warranted to begin to decipher the complex nature of these primitive enzymes.

## 10. Conclusions

The increase in atmospheric and oceanic O_2_ billions of years ago was a critical step in the progression of cellular life, and the accumulation of this vital gas accelerated the size, complexity, and development of intricate features in primitive species. It is undeniable that O_2_ is a Janus-faced molecule with the ability to be both useful and harmful in its reactions. The SOD family of enzymes is one of the most primitive class of functional proteins that have been described, and its convergent mechanism across its various isoforms depicts its importance over the evolutionary history of modern life. While exhaustive studies have been performed in the examination of these enzymes, many questions still exist. Why is there only one isoform of SOD found in specific subcellular compartments if it is so important? Why is there no compensation of the other isoforms of SOD when one is lost? How are modern-day species able to survive without a specific isoform of SOD? How does SOD specifically contribute to redox signaling? These and many other enigmas still remain, which truly illuminates the ever-expanding complexity of redox biology that is more than simply oxidative stress.
